# Rapid detection of *Salmonella enterica* serovar Typhimurium DT104 strains by the polymerase chain reaction

**DOI:** 10.1186/s13028-015-0143-x

**Published:** 2015-09-25

**Authors:** Shoichiro Yukawa, Yutaka Tamura, Kiyoshi Tanaka, Ikuo Uchida

**Affiliations:** Department of Comparative Animal Science, College of Life Science, Kurashiki University of Science and The Arts, 2640 Tsurajimachou Nisinoura, Kurashiki-shi, Okayama, 712-8505 Japan; Department of Veterinary Medicine, School of Veterinary Medicine, Rakuno Gakuen University, 582 Bunkyoudaimidorimachi, Ebetsu-shi, Hokkaido 069-8501 Japan; Hokkaido Research Station, National Institute of Animal Health, 4 Hitsujigaoka, Toyohira, Sapporo, 062-0045 Japan; United Graduate School of Veterinary Sciences, Gifu University, 1-1 Yanagido, 15 Gifu-shi, Gifu, 501-1193 Japan

**Keywords:** Detection, Epidemiologic investigation, PCR, *Salmonella* Typhimurium DT104, ST104

## Abstract

**Background:**

Infection with *Salmonella enterica* is a major public health concern in developed countries, and multidrug-resistant strains have become increasingly prevalent. *S. enterica* serovar Typhimurium DT104 (DT104) strains are prevalent in livestock in Japan and include numerous strains of multidrug-resistant *S. enterica*. Epidemiological analysis of these strains is critical for both agriculture and public health; however, diagnostic tests for these strains have yielded inconsistent results.

**Results:**

We developed a rapid, simple, and inexpensive polymerase chain reaction test to detect multi-drug resistant DT104 strains. We designed primers specific to the prophage ST104 sequence encoded by DT104 strains and assessed the specificity of these primers by assaying a panel of 50 *S.enterica* isolates. Amplification products of the expected size were generated from the genomes of each of the DT104 strains; however, the ST104 primers failed to amplify products from non-DT104 strains of *S.enterica* serovar Typhimurium or other *S. enterica* serovars. Furthermore, a probe generated using the ST104 primers detected a restriction fragment encoding the ST104 region of DT104 by Southern hybridization.

**Conclusions:**

The ST104 primers exhibit specificity to DT104 strains and are suitable for epidemiological applications.

## Background

Infection with *Salmonella enterica* is a major public health concern in developed countries. The multidrug-resistant *S. enterica* serovar Typhimurium (*S. enterica* Typhimurium) definitive phage type 104 (DT104) strain was first isolated in the late 1980s and has since spread across Japan and through numerous western countries [1–5]. DT104 strains exhibit a core pattern of resistance to ampicillin, chloramphenicol, streptomycin, sulfonamides, and tetracycline; the genes that confer resistance to these antimicrobials are encoded by a chromosomal locus containing Class 1 integrons [6]. Malthe et al. [7] recently reported that DT104 strains are also resistant to ciprofloxacin, making them particularly difficult organisms to control.

DT104 strains have a wide host range and are pathogenic in humans and livestock [8], with cattle, in particular, being considered a major reservoir. Although food-borne illnesses associated with *S. enterica* Typhimurium decreased in Japan during the 1990s [9], human nontyphoidal salmonellosis caused by DT104 strains remains a serious public health problem [10]. In a previous study, Sameshima et al. [11] demonstrated that DT104 strains have existed in Japanese livestock since 1990 and found that more than half (36 of 68) of the *S. enterica* Typhimurium isolates resistant to five or more antibiotics were DT104 strains. Furthermore, Esaki et al. [12] reported several pulsotypes of DT104 in multiple animal species in Japan. These findings suggest that, rather than evolving from a single clonal strain, multiple DT104-related strains have been introduced in Japan through various routes, including domestic animals, wild birds, and/or food.

Japanese DT104 isolates are genetically similar to the predominant strains found abroad, and the previous studies have demonstrated that all DT104 isolates contain the same prophage (designated ST104) [10–14]. However, Hermans et al. [15] identified strains that contain additional prophages (ST104B and/or ST64B) that are similar to ST104 but represent distinct DT104 subtypes. While epidemiological investigation of DT104 strains is an important task in agriculture and public health, the identification of *S. enterica* Typhimurium phage types is time-consuming and requires specially trained personnel [16]. In addition, possession of this phage type is limited to a few centralized laboratories. Several reports have described PCR detection of DT104 [17, 18]; however, these assays are inaccurate, often exhibiting nonspecific and false-positive reactions. In this study, we describe an improved PCR-based method for detecting DT104 strains.

## Methods

### Polymerase chain reaction (PCR) assays

**Fig. 1:**
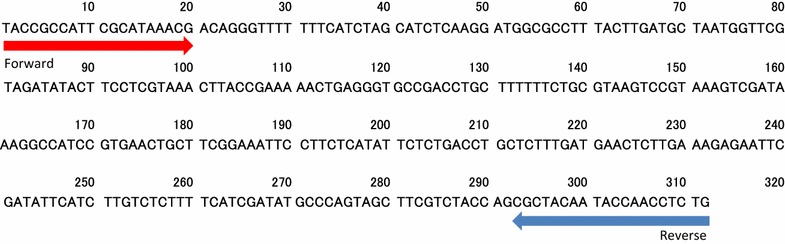
Nucleotide sequence of the ST104 *erf*-like gene. The ST104 sequence was amplified from *Salmonella enterica* serovar Typhimurium DT104 by using the ST104 primers

The ST104forward and ST104reverse primers were designed to amplify a 312-base pair (bp) segment of the *erf*-like gene within the ST104 sequence (Fig. 1; Table 1) and were used to detect *S. enterica* Typhimurium DT104 strains. For comparison, we used the DT104F and DT104R primers [17], which amplify a 162-bp cryptic sequence from DT104 strains (Table 1). In addition, primers InvAforward and InvAreverse, which amplify a 512-bp segment of the *S. entericainvA* gene [15, 19, 20], were used as positive controls for sample preparation and amplification (Table 1).

**Table 1:** Primer sequences and expected PCR product sizes

Primer	Nucleotide sequence (5′–3′)	Product size (bp)
ST104F	TACCGCCATTCGCATAAACG	312
ST104R	CAGAGGTTGGTATTGTAGCG	
DT104F	GTCAGCAGTGTATGGAGCGA	162
DT104R	AGTAGCGCCAGGACTCGTTA	
InvA-F	TTGTTACGGCTATTTTGACCA	521
InvA-R	CTGACTGCTACCTTGCTGATG	

*bp* base pairs

The primers used for PCR analysis of 50 *S. enterica* isolates are listed in Table 2. The isolates included 30 *S. enterica* Typhimurium DT104 isolates; 12 non-DT104 *S. enterica* Typhimurium isolates; two isolates of *S. enterica* Enteritidis; and one isolate each of *S. enterica* Brandenburg, *S. enterica* Dublin, *S*. *enterica* Javiana, *S. enterica* Montevideo, *S*. *enterica* Nagoya, and *S. enterica* Saintpaul. The *Salmonella* strains were obtained from the National Veterinary Assay Laboratory in Japan (Table 2). The clinical isolates were epidemiologically unrelated. After serological examination, the serovars and phage types were identified. To harvest template DNA, *S. enterica* strains were grown overnight at 37 °C on brain–heart infusion agar plates and then one loopful of cells of each strain was boiled in 200 µl sterile distilled water for 10 min. After centrifugation, the supernatants were stored at −20 °C. PCR amplification reactions were performed as follows: 1 μl template was added to a 20 μl reaction mixture containing 1× PCR buffer (Takara Bio, Tokyo, Japan), 1 μM of each primer, 200 μM deoxynucleoside triphosphates, 2 mM MgCl_2_, and 0.5 U of AmpliTaq DNA Polymerase (Takara Bio). Amplification was performed in 0.2 ml tubes in an iCycler PCR Detection System (Bio-Rad Laboratories, Hercules, CA, USA) as follows: denaturation at 95 °C for 1 min; 30 cycles of 94 °C for 30 s, 55 °C for 30 s, and 72 °C for 1 min; and a final extension cycle at 72 °C for 3 min. The reaction mixtures were separated by 2 % agarose gel electrophoresis (Agarose L03; Takara Bio).

**Table 2:** Compiled results of PCR analyses of *Salmonella enterica* strains

Serotype	Phage type	No. of strains	No. of PCR positive reactions (%)		
			ST104	DT104	invA
*S.* Typhimurium	DT104	30	30 (100)	29 (97)	30 (100)
	Non-DT104^a^	12	0 (0)	0 (0)	12 (100)
*S.* Brandenberg		1	0 (0)	0 (0)	1 (100)
*S.* Dublin		1	0 (0)	0 (0)	1 (100)
*S.* Enteritidis		2	0 (0)	0 (0)	2 (100)
*S.* Javiana		1	0 (0)	0 (0)	1 (100)
*S.* Montevideo		1	0 (0)	1 (100)	1 (100)
*S.* Nagoya		1	0 (0)	0 (0)	1 (100)
*S.* Saintpaul		1	0 (0)	1 (100)	1 (100)

^a^Non-DT104 denotes *S. enterica* Typhimurium strains lacking phage type 104

### Pulsed-field gel electrophoresis

Pulsed-field gel electrophoresis (PFGE) was performed by contour-clamped homogeneous electric-field electrophoresis using a CHEF-DR^®^ II system (Bio-Rad). PFGE was used in combination with Southern blotting to achieve high resolution when detecting ST104 primer-targeted sequences. One of the DT104 strains was grown overnight at 37 °C in lysogeny broth. Cells were harvested by centrifugation for 10 min at 3600×*g* and resuspended in 0.5 ml of NT buffer [10 mM Tris–HCl (pH 7.5), 1 M NaCl]. An aliquot (0.3 ml) of each suspension was transferred to a microcentrifuge tube, and the cells were pelleted at 12,000×*g* and washed twice in NT buffer. The cell suspensions were mixed with an equal volume of 1.5 % low-melting point agarose (FMC Bioproducts, Philadelphia, PA, USA) and allowed to solidify in 100 µl plug molds (Bio-Rad). The agarose plugs were incubated overnight at 55 °C in 1 ml lysis buffer [60 mM Tris–HCl (pH 7.5), 50 mM ethylenediaminetetraacetic acid (EDTA), 1 % sodium lauroyl sarcosine, lysozyme (1 mg/ml), RNase (1 µg/ml), proteinase K (1 mg/ml)], washed twice for 30 min with TE buffer [10 mM Tris–HCl (pH 7.5), 0.1 mM EDTA] containing the protease inhibitor phenylmethylsulfonyl fluoride (1 mM), and washed four additional times for 30 min each with 1 ml of TE buffer. A slice of each plug was cut and incubated in 400 µl restriction buffer containing 50 U *Xba*I (Takara Bio) at 37 °C for 2 h. The restriction enzyme-digested DNA fragments were separated using pulsed-field electrophoresis-certified agarose (Bio-Rad). Electrophoresis was performed for 25 h at 14 °C and 6 V/cm in twofold-diluted TBE buffer with pulse times of 5–80 s. Lambda PFGE DNA markers (New England BioLabs, Ipswich, MA, USA) were used as DNA size markers.

### Southern hybridization

A DNA fragment containing the ST104 bacteriophage region was amplified from DT104 with the ST104F and ST104R primers (Table 1). The PCR product was purified using a QIAquick^®^ Gel Extraction Kit (Qiagen, Hilden, Germany) and labeled with digoxigenin (DIG)-11-deoxyuridine triphosphate by random priming using a DIG-High Prime DNA Labeling Kit (Boehringer GmbH, Germany), according to the manufacturer’s instructions. The labeled PCR product was then used as a probe for Southern hybridization analyses. After PFGE of agarose plugs, genomic DNA from *S. enterica* strains was transferred to positively charged membranes (Boehringer) by capillary action. Prehybridization (>30 min) and hybridization (>16 h) were performed in DIG Easy Hyb Solution (Boehringer) under high-stringency conditions, and hybrid detection was achieved with a DIG Luminescent Detection Kit (Boehringer), according to the manufacturer’s instructions. Hybridization products were detected by exposing Hyperfilm MP (Amersham International, Little Chalfont, UK) to the membranes for 1–10 min at room temperature. Films were developed in a Kodak X-Omat processor (Rochester, NY, USA).

### ST104 primer target analysis

BLAST was used for in silico identification of genes with strong homology to the sequence targeted by the ST104 primers (Fig. 1).

## Results

### ST104 primers are specific for DT104 strains

**Fig. 2:**
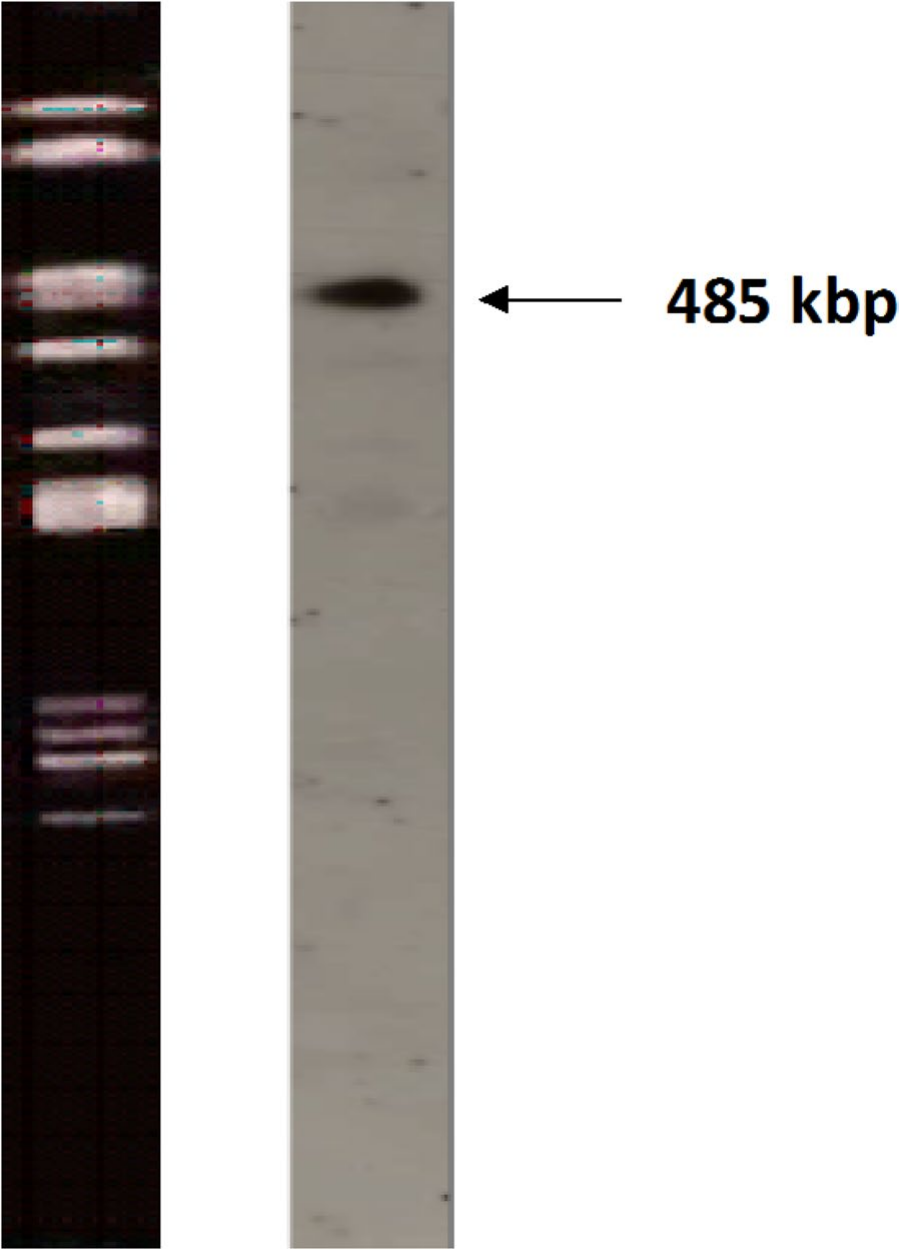
Southern blot hybridization analysis of *Salmonella enterica* strains. The ST104 sequence was amplified from *S. enterica* serovar Typhimurium DT104 with the ST104 primers and used as a probe for Southern blot analysis of *S. enterica* strains. A representative image of the fingerprinting pattern of DT104 after restriction digestion with *Xba*I is depicted in the *left lane*. The result of a subsequent Southern blot hybridization experiment, using the digoxigenin (DIG)-11-deoxyuridine triphosphate-labeled ST104 PCR fragment, is depicted in the *right lane*

To assess the specificity of the ST104 primers, we performed PCR analysis with genomic DNA from a panel of 50 *S. enterica* isolates (Table 2). The previously published DT104 primers [17] were used for comparison. While the ST104 primers amplified the expected 312-bp product from each of the 30 DT104 strains tested, PCR yielded no products from the 12 non-DT104 *S*. *enterica* Typhimurium strains (Table 2). Likewise, the ST104 primers failed to amplify a product from eight *S. enterica* serovars (Table 2). Conversely, the DT104 primers amplified the expected 162-bp product from 29 of the DT104 strains (97 %). Although this 162-bp product was not detected in PCR reactions with non-DT104 *S*. *enterica* Typhimurium strains, nonspecific amplification was detected for one DT104 strain and in all of the non-DT104 strain reactions. Furthermore, the DT104 primers yielded false-positive products from the *S*. *enterica* Montevideo and *S*. *enterica* Saintpaul genomes (25 % of the non-Typhimurium serovars tested) and nonspecific products from the DNA of *S*. *enterica* Javiana, *S*. *enterica* Nagoya, *S*. *enterica* Brandenburg, and *S*. *enterica* Enteritidis (50 %; Table 2; Fig. 2). The InvA primers, which were used as positive controls for sample preparation and amplification, yielded the expected product from all 50 *S. enterica* strains (Table 2).

### Detection of the ST104 region in DNA fragments from DT104 strains by Southern hybridization

To detect the ST104 sequence in DT104 isolates, we performed PFGE followed by Southern hybridization. The Southern blot probe was generated by amplifying the ST104 region from DT104 DNA with the ST104 primers. The ST104 probe hybridized to a region within a genomic DNA restriction fragment of approximately 485 kilobase pairs (kbp) in the DT104 strain (Fig. 2).

### In silico analysis of target sequences for the ST104 primers

A 312-bp sequence targeted by the ST104 primers was detected from two non-DT104 *Salmonella* Typhimurium strains, one *Salmonella* Pullorum strain, and a bacteriophage [GenBank: CP003836, CP006575, AP011957, and JF900176]. In addition, strong sequence homology above 99 % (310/312) was detected in the *S. enterica* subsp. *arizonae* strain [GenBank: CP000880].

## Discussion

We aimed to develop a rapid and specific screen for *S*. *enterica* serovar Typhimurium DT104. To this end, we developed primers to detect the temperate phage ST104, which is encoded by all DT104 isolates. Because ST104 exhibits a high level of sequence identity to *Enterobacteria phage P22*, which also exists in *S. enterica* Typhimurium, the ST104 primers were carefully designed to avoid unintended detection of this phage. Indeed, our results demonstrate that these primers exhibited a high level of sensitivity for DT104 strains, and did not result in false positives or negatives. Moreover, our primers were more sensitive and specific than the previously published DT104 primers [17], which also elicited several false-positive and nonspecific reactions. These results indicated that PCR analysis using the ST104 primers is a reliable and valuable diagnostic tool for the rapid identification of DT104 strains. In addition, we utilized the ST104 primers to generate a probe for Southern blot analysis of DT104 strains. This probe specifically hybridized to a restriction fragment (approximately 485 kbp) present in DT104 strains. While BLAST sequence analysis of the ST104 primer targets showed homology with a few non-DT104 *Salmonella* strains, the ST104 primers did not result in false-positive or -negative results with *Salmonella* strains in this study.

## Conclusions

PCR analysis with ST104 primers is an inexpensive, rapid, and simple technique for detecting DT104 strains in epidemiological applications. While gene sequences similar to ST104 may exist in non-DT104 strains of *S. enterica* Typhimurium, our PCR method may facilitate future studies endeavoring to screen for specific phage types.
